# The Combination of Beta-Blockers and ACE Inhibitors Across the Spectrum of Cardiovascular Diseases

**DOI:** 10.1007/s10557-021-07248-1

**Published:** 2021-09-17

**Authors:** Martin H. Strauss, Alistair S. Hall, Krzysztof Narkiewicz

**Affiliations:** 1 University of Toronto, North York General Hospital, Toronto, ON Canada; 2 Leeds School of Medicine, University of Leeds, Leeds, UK; 3 Department of Hypertension and Diabetology, Faculty of Medicine, Medical University of Gdansk, Debinki 7c, 80-952 Gdansk, Poland

**Keywords:** Beta-blockers, Renin angiotensin aldosterone system, Angiotensin-converting enzyme inhibitors, Angiotensin II receptor blockers, Hypertension, Coronary artery disease

## Abstract

Cardiovascular disease is the leading cause of mortality worldwide, affecting a wide range of patients at different stages across the cardiovascular continuum. Hypertension is one of the earliest risk factors in this continuum and can be controlled in most patients with currently available antihypertensive agents. However, goals are often not met because treatments are not optimized in terms of tailoring therapy to individual patients based on their hypertension subclass and cardiovascular risk profile and initiating early use of adapted-dose, single-pill combinations. In this context, beta-blockers in combination with angiotensin-converting enzyme (ACE) inhibitors are of special interest as a result of their complementary actions on the sympathetic nervous system and renin–angiotensin–aldosterone system, two interlinked pathways that influence cardiovascular risk and disease outcomes. In addition to their antihypertensive actions, beta-blockers are used to manage arrhythmias and treat angina pectoris and heart failure, while ACE inhibitors provide cardioprotection in patients with acute coronary syndromes and treat congestive heart failure. A broad range of patients may therefore receive the combination in routine clinical practice. This paper examines the supporting evidence for beta-blockers and ACE inhibitors in each of the above indications and considers the rationale for combining these agents into a single pill, using data from bisoprolol and perindopril randomized controlled trials as supporting evidence. Combining these established antihypertensive agents into a single pill continues to provide effective blood pressure lowering and improved cardiovascular outcomes while allowing a greater proportion of patients to rapidly achieve treatment targets.

## Introduction

Cardiovascular disease (CVD) is the leading cause of mortality worldwide, and its prevention is therefore a major public health priority [1, 2]. CVD represents the culmination of continuous exposure to cardiovascular risk factors, with a progressively worsening pathogenesis that continues for decades. The gradual development of atherosclerotic lesions combined with other risk factors leads to the spectrum of cardiovascular diseases, including angina pectoris, myocardial infarction, chronic heart failure, and death. Intervention at any point along the continuum can modify CVD progression, but even with a range of evidence-based clinical guidelines and effective interventions, the majority of patients do not achieve sufficient risk factor control [3]. A strong focus of guidelines published recently has therefore been for simple and consistent recommendations with an emphasis on strategies that use available treatments more effectively [4, 5]. This includes a focus on how to optimize treatment by prescribing effective combinations as single pills and tailoring treatment to individual patients based on their cardiovascular risk profile and position in the CVD continuum.

Two neurohormonal systems, the sympathetic nervous system (SNS) and renin–angiotensin–aldosterone system (RAAS), are intricately involved in the progression of disease throughout the CVD continuum. Furthermore, the two systems interact in a positive-feedback manner whereby sympathetic activation results in increased renin secretion leading to activation of the RAAS, and RAAS activation leads to sympathetic overactivity by increasing noradrenaline release [6]. There is therefore considerable rationale for combining pharmacological therapies that target both neurohormonal pathways.

**Fig. 1:**
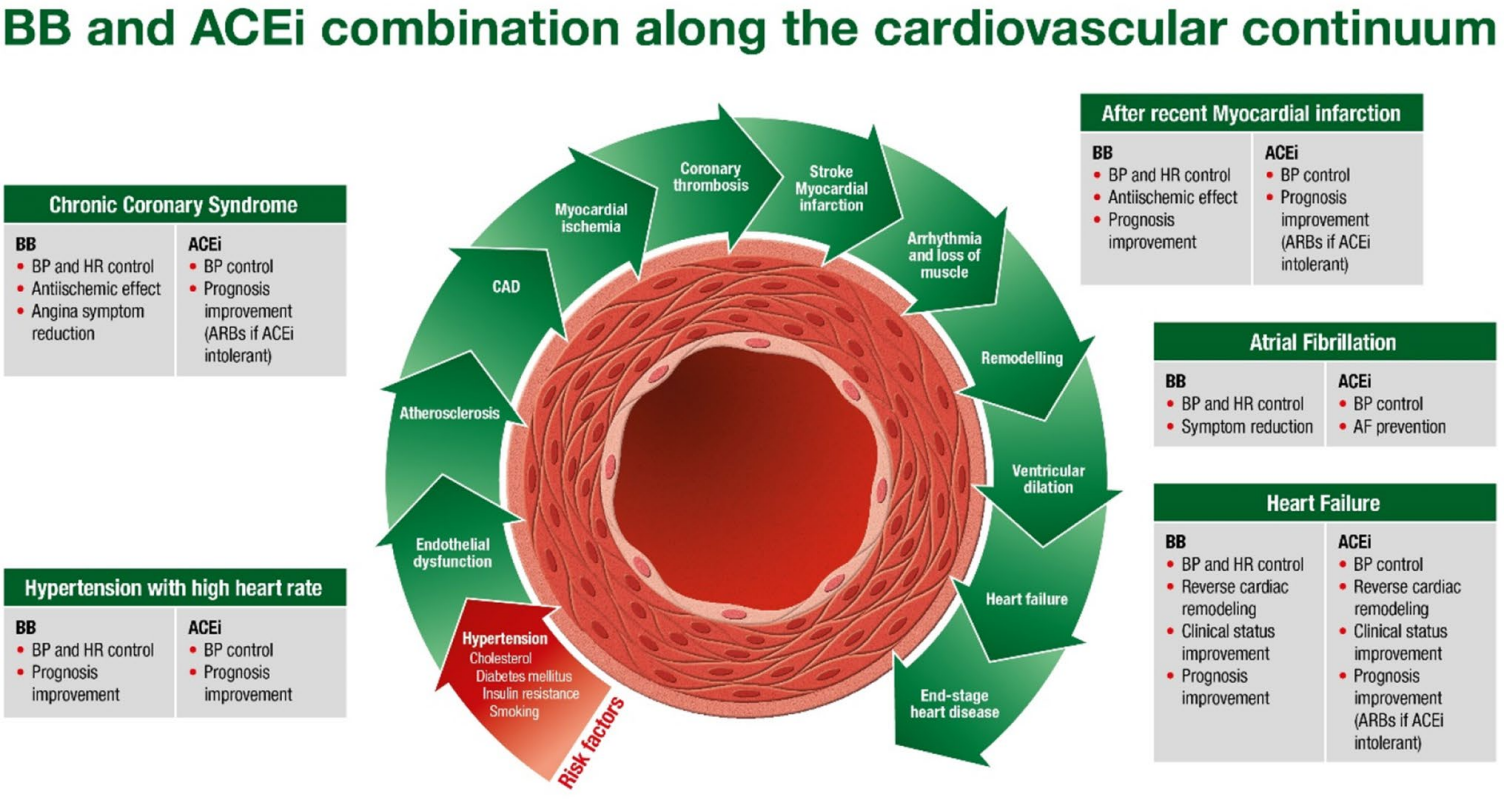
Beta-blocker and ACE inhibitor combination along the cardiovascular continuum (adapted with permission from Dzau and Braunwald, 1991 [104], and Fox, 2007 [105]). ACEi, angiotensin-converting enzyme inhibitors; ARB, angiotensin receptor blockers; BB, beta-blockers; BP, blood pressure; HR, heart rate

This paper examines the rationale for combining a beta-blocker with an angiotensin-converting enzyme (ACE) inhibitor, at each stage of the CVD continuum, from individuals with cardiovascular risk factors to patients with chronic heart failure (Fig. 1). For each indication (patients with hypertension, coronary artery disease (CAD), atrial fibrillation (AF), and heart failure), the supporting evidence for beta-blockers and ACE inhibitors is presented. The combination of the beta-blocker bisoprolol and ACE inhibitor perindopril will then be discussed.

## Hypertension

Hypertension is one of the earliest risk factors in the CVD continuum and remains involved at each subsequent stage. Recently, guidelines for the management of arterial hypertension have lowered blood pressure (BP) targets [4, 5]. The ESC/ESH 2018 guidelines consider everyone with a BP greater than 140/90 mmHg, a candidate for antihypertensive treatment, with the objective of lowering systolic BP (SBP) to a range of 130 to < 140 mmHg within 3 months; if well tolerated, this can be further lowered to a BP range of 120 to < 130 mmHg in most patients [4]. The AHA/ACC 2017 guidelines applied a treatment target of < 130/80 mmHg [5]. However, results from the latest EUROASPIRE survey, which focused on asymptomatic individuals in primary care at high risk of developing CVD, found that only 42% of individuals achieved their recommended BP goals despite high use of antihypertensive medications [7]. Similarly, recent estimates from the USA indicate that 44% of adults taking antihypertensive medication have uncontrolled BP [8]. The importance of BP control is highlighted in another US study, which found that treated but uncontrolled hypertensives had increased risk of all-cause and CVD mortality compared to normotensives [9].

By targeting multiple mechanisms, initial combination treatment with at least two antihypertensive agents offers a number of benefits over doubling the dose of monotherapy including greater reductions in BP [10], greater protection against hypertension-mediated organ damage (HMOD) [11, 12], fewer adverse events, and thereby increased patient adherence, all of which will culminate in reduced physician inertia regarding uptitration [13, 14]. This has been addressed by current guidelines, which now with few exceptions, other than low-risk grade I hypertension and the frail elderly, recommend a single-pill combination of two antihypertensive agents first-line [4, 5]. ACE inhibitors and beta-blockers represent two of the five main drug classes recommended for the treatment of arterial hypertension. The following sections will summarize the evidence supporting the use of these agents in a range of hypertension subtypes.

### Evidence Supporting ACE Inhibitors

In the 2018 ESC/ESH guidelines for the management of arterial hypertension, initial dual treatment combinations comprise an ACE inhibitor or angiotensin receptor blocker (ARB) with either a calcium channel blocker (CCB) or thiazide/thiazide-like diuretic [4].

ACE inhibitors are indicated for uncomplicated hypertension, as well as for hypertension and concomitant CAD (including post-myocardial infarction), chronic kidney disease (CKD), type 2 diabetes, heart failure with reduced ejection fraction, or atrial fibrillation [4].

ACE inhibitors, ARBs, and CCBs are more effective than beta-blockers or diuretics at regressing left ventricular hypertrophy (LVH) for an equivalent reduction in BP [4, 15, 16]. Reversal of LVH, which represents a high-risk phenotype for the development of AF, has been shown to be associated with a substantial decrease in fatal and non-fatal cardiovascular complications, including new onset AF [17].

Both ACE inhibitors and ARBs reduce albuminuria more than other BP-lowering drugs and are effective at delaying the progression of diabetic and non-diabetic CKD. They are also the only antihypertensive agents for which evidence is available of a reduced risk of end-stage renal disease [18]. In the AHA/ACC 2017 hypertension guidelines, an ACE inhibitor is the preferred drug if albuminuria is present, although an ARB can be used in case of ACE inhibitor intolerance [5]. A recent meta-analysis of 119 randomized controlled trials in patients with CKD [19] found that ACE inhibitors compared to ARBs were associated with higher probabilities of reducing kidney failure and cardiovascular death and that ACE inhibitors, but not ARBs, reduced all-cause death compared to control.

In patients with type 2 diabetes, the ESC/ESH 2018 guidelines recommend initiation of a two-drug regimen combining a RAAS inhibitor, which have been shown to reduce albuminuria and the appearance or progression of diabetic nephropathy compared with other antihypertensive agent classes [20], with either a CCB or thiazide/thiazide-like diuretic [4]. The UK National Institute for Health and Care Excellence (NICE) recommends an ACE inhibitor as first-line antihypertensive in patients with type 2 diabetes with ARBs reserved for those in whom ACE inhibitors are not tolerated [21]. While the BP-lowering efficacy of RAAS inhibitors is reduced in those of black African origin due to a greater likelihood of having a low renin profile, the rationale for their use remains high because of the frequent prevalence of comorbid conditions, such as diabetes, cardiovascular disease, and CKD. The NICE guidelines therefore recommend starting a RAAS inhibitor simultaneously with either a CCB or diuretic [21].

ACE inhibitors and ARBs have similar BP-lowering efficacy, but there are no individual trial data or meta-analyses to show that ARBs reduce the risk of myocardial infarction or all-cause mortality [22]. In parallel meta-analyses of ACE inhibitor and ARB trials where the comparator has been a placebo, ARBs did not reduce mortality whatsoever (Table 1) [23]. In a meta-analysis of contemporary hypertension trials, ACE inhibitors vs. all comparators reduced all-cause mortality by 10% (*P* < 0.05), whereas ARBs in a parallel meta-analysis were associated with no reduction in mortality. The mortality reduction with ACE inhibitors was “above” and independent of BP-lowering [24]. ARBs had no such effect. A Cochrane meta-analysis that compared ACE inhibitors with ARBs in primary hypertension recommended ACE inhibitors as the preferred RAAS inhibitor and stated, “while ARBs are slightly better tolerated than ACE inhibitors, there is a higher quality of data supporting the use of ACE inhibitors to prevent death, strokes, and heart disease that must be considered before choosing ARB over ACE inhibitors” [25].

**Table 1:** Risk of myocardial infarction, cardiovascular mortality, and all-cause mortality in parallel meta-analyses of placebo-controlled trials of angiotensin-converting enzyme inhibitors and angiotensin receptor blockers ( reproduced with﻿ permission from Strauss and Ha﻿ll, 2017 [23])

	**ACEi vs. placebo**				**ARB vs. placebo**			
	**MI**	**CV death**	**All-cause death**	***N***	**MI**	**CV death**	**All-cause death**	***N***
High risk, Bangalore et al. [103]	0.83 (0.78–0.9)	0.83 (0.7–0.99)	0.89 (0.80–1.0)	62,398	0.93 (0.85–1.03)	1.02 (0.92–1.14)	1.01 (0.96–1.06)	66,282
High risk, Savarese et al. [106]	0.81 (0.75–0.88)	0.9 (0.78–1.03)	0.91 (0.85–0.98)	53,791	0.9 (0.8–1.02)	1.03 (0.85–1.26)	1.01 (0.94–1.08)	54,421
Diabetes mellitus, Cheng et al. [107]	NA	0.83 (0.70–0.99)	0.89 (0.79–0.99)	21,997	NA	1.21 (0.81–1.8)	1.03 (0.89–1.18)	13,304
Hypertension, Thomopoulos et al. [108]	NA	0.87 (0.78–0.98)	0.91 (0.85–0.98)	49,440	NA	1.03 (0.94–1.13)	1.01 (0.97–1.06)	65,256

Values indicate hazard ratio (95% confidence interval). *ACEi* angiotensin-converting enzyme inhibitor, *ARB* angiotensin II receptor blocker, *CV* cardiovascular, *MI* myocardial infarction

### Evidence Supporting Beta-Blockers

#### Blood Pressure Control and Cardiovascular Outcomes

While all hypertension guidelines recommend an initial combination of an ACE inhibitor/ARB with either a CCB or diuretic, beta-blockers may form part of the combination at any stage of treatment when they are specifically indicated, i.e., when a second comorbidity (CAD, symptomatic angina, myocardial infarction, heart failure, atrial fibrillation) is present [4]. A meta-analysis of 12 trials found that beta-blocker use for the treatment of hypertension may be associated with an increased risk for new-onset type 2 diabetes, primarily in patients with the metabolic syndrome [26], and their use in these patients would therefore not be recommended unless the benefits outweighed the risks. Randomized controlled trials and meta-analyses have demonstrated that when compared with placebo, beta-blockers significantly reduce the risk of stroke, heart failure, and major cardiovascular events in hypertensive patients. When compared with other BP-lowering drugs, beta-blockers are usually equivalent in preventing major cardiovascular events. The exception is stroke, for which a Cochrane Database analysis demonstrated a higher incidence in patients whose antihypertensive treatment was initiated with a beta-blocker compared with a RAAS inhibitor or a CCB [27]. The apparent reduced benefit in stroke, however, must be viewed in the context of the respective trials. In the meta-analysis of beta-blocker vs. CCB, 95% of the patients were from two trials (ASCOT [28] and INVEST [29]) with atenolol as the beta-blocker in both trials. Atenolol is administered once a day despite a half-life of as little as 6 h and thus would be unlikely to prevent the early morning surge of BP that is associated with an increase in the risk of stroke. The stroke observation may originate from small differences in achieved BP (including central SBP between different drug treatments), to which cerebrovascular events may be especially sensitive [4]. In the meta-analysis of beta-blocker vs. RAAS inhibitors, more than 90% of the patients were from the LIFE study (losartan vs. atenolol) [30] and therefore subject to atenolol’s known limitation as discussed above. In the LIFE trial, losartan did achieve a 1 mmHg lower SBP. However, in the atenolol arm of LIFE, there was an absolute 1% higher incidence of each of atrial fibrillation, smoking, and isolated systolic hypertension at baseline. These are all important risk factors for stroke, thus favoring a lower risk of stroke in the losartan arm. In the Cochrane meta-analysis, CCB but not RAAS inhibitors had a mortality benefit compared to beta-blockers [27]. However, a recent meta-analysis by Thomopoulos et al. [31] concluded that beta-blockers exhibit a substantial risk-reducing ability for all events when prescribed to lower BP and therefore can be used as additional agents in patients with hypertension.

The Conduit Artery Functional Endpoint (CAFÉ) substudy of the ASCOT trial showed that beta-blockers, specifically atenolol, were less effective at controlling central SBP as compared with peripheral brachial BP, the former being more strongly related to vascular disease and outcome than brachial BP [32, 33]. Beta-blockers differ in their pharmacological mechanisms of action, and while there is little evidence to support the use of one agent over another for lowering BP, physicians should use the pharmacological diversity of these agents and the clinical characteristics of patients to individualize treatment and optimize care. A randomized controlled study on the effects of bisoprolol and atenolol in 109 treatment-naïve patients with hypertension found that both drugs similarly reduced brachial BP, but bisoprolol was significantly more effective at decreasing central systolic BP and aortic pulse pressure [34]. The lesser effect of atenolol on central systolic BP combined with its relatively short duration of action compared with other agents could explain most of the observed differences in outcome in atenolol-based trials. By reducing central systolic BP, bisoprolol helps to avoid adverse effects related to increased central aortic pressure.

The SNS is involved in multiple homeostatic functions including energy balance and BP control [35, 36]. Patients with hypertension and resting heart rate > 80 bpm are characterized by marked sympathetic overactivity and higher cardiovascular risks compared with those with a heart rate < 80 bpm [37, 38]. This was eloquently demonstrated in a trial of 193 patients with untreated moderate hypertension [37]. In patients with similar clinic and ambulatory BP, those with heart rates > 80 bpm had a more active sympathetic nervous system as evaluated by both venous norepinephrine and microneurographic assessment of sympathetic muscle nerve traffic—with the latter being a more sensitive measure of sympathetic activity and almost a linear correlation to heart rate. The higher the heart rate, the greater was the left ventricular mass index, which appears to have been driven by over activation of the SNS. Similar correlations have been found with traditional cardiovascular risk factors such as hypertension, dyslipidemia, diabetes, and obesity and is a powerful predictor of cardiovascular outcomes [39, 40]. In the VALUE trial, individuals with an elevated heart rate remained at risk of cardiovascular events even if their BP was well controlled [40], suggesting that for optimal risk reduction, both BP and heart rate must be lowered. These findings have led to elevated heart rate being listed among the factors influencing cardiovascular risk in patients with hypertension [4] and provide a strong rationale for the use of interventions that target heart rate by modulating the SNS [41].

Optimal control of hypertension caused by sympathetic overdrive requires a beta-blocker component to the combination therapy. The use of a selective beta-1 blocker will inhibit sympathetic activity in the heart and kidney, preserve beta-2-mediated vasodilation, and reduce the risk of adverse effects mediated by blockade of beta-2 receptors in the lungs and peripheral tissues [4].

An open-label comparison of bisoprolol with metoprolol in 186 Chinese patients with mild-to-moderate hypertension in the CREATIVE study showed that while both agents lowered mean heart rate in the last 4 h of the dosing period, bisoprolol led to a significantly greater reduction with a comparable BP response [42]. However, while heart rate control throughout the dosing period may be an important consideration when selecting a long-acting medication for patients with hypertension, studies to date have not been able to show that lowering heart rate in patients with hypertension improves cardiovascular outcomes [43].

## Coronary Artery Disease

Data from the World Health Organization indicate that CAD remains the single largest cause of death worldwide [44]. It is also responsible for a major burden of disease, rated in the top five leading causes of disability-adjusted life-years (DALYs) worldwide [45]. CAD occurs when there is an inadequate blood supply to the myocardium, usually as a result of atherosclerotic build-up in the coronary arteries. It encompasses a spectrum of progressive disease with corresponding risk, e.g., obstructive vs. non-obstructive, single versus multi-vessel disease, and death. Clinical manifestations include angina pectoris (stable or unstable) and myocardial infarction. In patients with CAD, the goals of treatment are to reduce ischemia, improve quality of life, and prevent cardiovascular events and death.

### Evidence Supporting ACE Inhibitors

In recent ESC guidelines for the diagnosis and management of chronic coronary syndromes (CCS), ACE inhibitors are recommended for event prevention if a patient has other comorbid conditions (e.g., heart failure, hypertension, or diabetes) and should be considered in CCS patients at very high risk of cardiovascular events [46].

ACE inhibitors can reduce mortality, myocardial infarction, stroke, and heart failure among patients with left ventricular dysfunction, previous vascular disease, and high-risk diabetes [46].

Three large trials have assessed the effect of ACE inhibitors in patients with stable CAD: HOPE, EUROPA, and PEACE. In the HOPE trial, which included 9297 patients with evidence of vascular disease or type 2 diabetes plus an additional cardiovascular risk factor, mean baseline BP was 139/79 mmHg, and the reduction in BP with ramipril was modest. Treatment with ramipril was associated with a 22% reduction in the composite endpoint of cardiovascular death, myocardial infarction, and stroke (*P* < 0.001) [47]. In the EUROPA trial, 12,218 patients with documented stable CAD without clinical evidence of heart failure were randomized to perindopril or placebo. The mean baseline BP of 137/82 mmHg was reduced by 5/2 mmHg in the perindopril arm. Over a mean follow-up of 4.2 years, the primary endpoint, a composite of cardiovascular death, myocardial infarction, or cardiac arrest, was observed in 8% of patients assigned to perindopril and 10% of patients assigned to placebo (relative risk reduction 20%; 95% CI 9–29%, *P* = 0.0003) [48]. There was also a trend towards a reduction (14%) in cardiovascular mortality and a significant 22% reduction in non-fatal myocardial infarction (*P* = 0.001). In the PEACE trial, 8290 patients with stable CAD and normal or slightly reduced left ventricular function were randomized to trandolapril or placebo [49]. Mean baseline BP was 133/78 mmHg and was reduced by 4.4/3.6 mmHg in the trandolapril arm. The PEACE trial found no difference between trandolapril and placebo in the incidence of the combined primary endpoint (a composite of death from cardiovascular causes, non-fatal myocardial infarction, or coronary revascularization) [49]. However, the inclusion of revascularization, which accounted for more than 85% of the primary endpoints in PEACE and which is physician dependent rather than drug related, may have diluted any treatment effect. In a meta-analysis of the HOPE, EUROPA, and PEACE trials, ACE inhibitors significantly reduced all-cause mortality compared with placebo. The composite outcomes of cardiovascular mortality, non-fatal myocardial infarction, or stroke occurred in 1599 (10.7%) of the patients allocated ACE inhibitor and in 1910 (12.8%) of those allocated placebo (odds ratio, 0.82; 95% CIs 0.76–0.88; *P* < 0.0001) [50]. All three trials recruited patients with normal baseline BP receiving background antihypertensive medication. The reductions in event rates with ACE inhibitor use were much greater than would have been expected for the modest decreases in BP achieved.

ACE inhibitors also have a number of benefits beyond their BP-lowering effects that bring value to the very high-risk population of patients with CAD. ACE inhibitors reduce angiotensin II levels, thus lowering BP, but also attenuate direct angiotensin-II-mediated tissue toxicity. They prevent the breakdown of bradykinin, which potentiates nitric-oxide-induced ischemic preconditioning, endothelial function, and fibrinolysis—all lifesaving attributes. Fibrinolysis is now recognized as a strong independent and novel marker of cardiovascular risk and a natural defense against arterial thrombotic occlusion [51, 52]. ACE inhibitors also counteract the vasoconstrictive effects that lead to increased oxidative stress, inflammation, and thrombosis [53].

A study based on data from a contemporary National Health Insurance claims database from South Korea analyzed patients undergoing PCI for either acute myocardial infarction (AMI, *n* = 21,747) or angina (*n* = 28,708). Patients were prescribed an ACE inhibitor or ARB at the time of discharge from hospital, which was at the discretion of the attending physician [52]. For the primary endpoint of all-cause death, the two groups were compared using a propensity-score matching analysis with a median follow-up of 2.2 years (interquartile range, 1.2–3.2). In the propensity-score matched AMI group (8341 pairs), the occurrence of all-cause death was significantly lower in the ACE inhibitor compared with ARB group (hazard ratio [HR] of ACE inhibitor 0.823; 95% confidence interval [CI]: 0.715–0.947; *P* = 0.006).

In a further observational study also from Korea, registry data were extracted for 11,288 patients with non-ST-segment elevation myocardial infarction (NSTEMI) who underwent percutaneous coronary intervention with drug-eluting stents and 2-year major clinical outcomes were compared between patients receiving beta-blockers plus ACE inhibitors with those receiving beta-blockers plus ARBs [54]. Although the cumulative incidences of all-cause death, cardiac death, target lesion revascularization, and non-target vessel revascularization were similar between the two groups, major adverse cardiac events (MACE), total revascularization rate, and target vessel revascularization rate were all significantly lower in the beta-blocker/ACE inhibitor group. A similar analysis with patients from the same registry with STEMI indicated that a beta-blocker/ACE inhibitor combination had a greater reduction on MACE than a beta-blocker/ARB combination [55].

ESC guidelines for the diagnosis and management of chronic coronary syndromes (CCS) recommend ACE inhibitors (or ARBs in case of ACE inhibitor intolerance) in patients at high cardiovascular risk because of coexisting conditions such as hypertension, LVEF ≤ 40%, diabetes, or CKD, but not for vascular protection per se [46].

### Evidence Supporting Beta-Blockers

Beta-blockers or CCBs are recommended as the first-choice anti-ischemic drugs, although no randomized controlled trial to date has compared this strategy to an alternative strategy using initial prescription of other anti-ischemic drugs, or the combination of a beta-blocker and a CCB. In patients with CAD, the dose of beta-blockers should be adjusted to limit heart rate to 55–60 bpm at rest [46].

Beta-blockers reduce myocardial workload and therefore oxygen consumption via a decrease in heart rate as well as BP. The reduction in heart rate allows for greater time spent in diastole, which increases perfusion of the ischemic myocardium. Beta-blockers are therefore of value during an acute coronary syndrome to reduce angina symptoms and in the case of a myocardial infarction to limit infarct size and reduce reinfarction risk.

In the TIBBS study (Total Ischaemic Burden Bisoprolol Study), 631 patients with stable angina and exercise stress tests showing electrocardiogram changes were randomized to the beta-blocker bisoprolol or the CCB nifedipine. After 8 weeks, total ischemic burden (measured by ST segment depression and duration) was reduced by 73% on bisoprolol and 46% on nifedipine [56]. Bisoprolol more effectively reduced the number and duration of transient ischemic episodes and the morning peak of ischemic activity.

In certain patients with recent myocardial infarction and those with chronic heart failure with reduced ejection fraction, beta-blockers have been associated with a significant reduction in mortality and/or cardiovascular events, but the protective benefit in patients with CAD without prior myocardial infarction or heart failure is less well established and lacks placebo-controlled trials. A retrospective analysis of 21,860 matched patients from the REACH (REduction of Atherothrombosis for Continued Health) Registry showed no reduction in cardiovascular mortality with beta-blockers in patients with either CAD with risk factors only, known prior myocardial infarction, or known CAD without myocardial infarction [57].

A recent analysis of data from the UK Clinical Practice Research Datalink (CPRD) compared the incidence and risk of mortality and CVD events in patients with angina receiving monotherapy with bisoprolol versus other beta-blockers or drugs other than beta-blockers in a primary care setting [58]. Treatment of new-onset angina with bisoprolol was associated with a significant reduction in the risk of mortality of at least 50% and reductions in CVD events of 23% for angina, 55% for myocardial infarction, and 39% for AF compared with other treatments, supporting its use as a first-line treatment for angina in primary care.

In the most recent ESC/ESH guidelines for the management of arterial hypertension [4], beta-blockers received a class I A level of evidence for use in hypertensive patients with a history of myocardial infarction based on a meta-analysis of 147 randomized trials of BP-lowering drugs that recorded CAD and stroke events [59]. The analysis demonstrated that beta-blockers were associated with a 29% reduction in CAD events (relative risk 0.71, 95% CI, 0.66 to 0.78) over and above their BP-lowering effect. This was significantly greater (*P* < 0.001) than the 15% reduction observed with other classes of drug in people with and without a history of CAD and in BB trials of patients without CAD. The risk reduction was observed for a few years after the infarct, after which time it reduced to that of other BP-lowering drugs [59].

## Atrial Fibrillation

AF is the most frequent cardiac arrhythmia and has been associated with a two- to threefold increased risk of cardiovascular mortality and stroke and fivefold risk of congestive heart failure [60, 61]. Experimental models suggest that hypertension may induce early and progressive changes in left atrial anatomy and function, which may promote AF through a variety of electrophysiological mechanisms [62]. There is substantial evidence to support aggressive risk factor modification for both primary prevention of AF and management of symptomatic AF. In addition to managing hypertension, diabetes, and sleep apnea, this includes following a healthy lifestyle (smoking cessation, limited alcohol intake, healthy diet, regular exercise) and losing weight if obese [63]. Oral anticoagulant therapy to prevent thromboembolism is also recommended in AF patients at risk of thromboembolic events [64]. The goals of medical therapy for patients with AF are to maintain sinus rhythm, avoid the risk of complications such as stroke and heart failure, and improve quality of life by minimizing symptoms. First-line treatment of AF is directed at controlling the ventricular rate with medications such as beta-blockers, non-dihydropyridine CCBs, or digoxin. Rate control is an integral part of the management of AF patients and is often sufficient to improve AF-related symptoms. If AF persists and the patient is still symptomatic from a functional capacity, consideration should be given to medical or electrical cardioversion to restore sinus rhythm, as well as pulmonary vein ablation in severely symptomatic patients.

### Evidence Supporting ACE Inhibitors

Hypertension is associated with LVH, systolic and diastolic dysfunction, increased left atrial pressure, hypertrophy and fibrosis, and a slowing of intra- and interatrial electrical conduction velocities [65]. Activation of the RAAS and in particular ACE and angiotensin II plays a key role in triggering these changes in cardiac structure and electrophysiology and thus to the development of AF. While all antihypertensive agents reduce left ventricular and left atrial filling pressures and thus wall stress, the prevention of structural changes such as atrial fibrosis may be an effect specific to RAAS blockers [66]. There is therefore a strong rationale for the use of RAAS blockers in patients with hypertension and AF for both primary prevention as well as to prevent AF recurrence.

However, to date, randomized controlled trials of RAAS blockers have failed to demonstrate an overall beneficial effect in the context of primary AF prevention [67, 68]. Most data on the role of RAAS blockers in patients with AF have come from post hoc analyses of large randomized trials and a number of meta-analyses. These have suggested that ACE inhibitors and ARBs reduce the incidence of new-onset AF in a variety of conditions, including hypertension [69, 70], left ventricular dysfunction [71, 72], and after coronary artery bypass graft surgery [73]. There was significant heterogeneity across the studies in terms of study populations, drugs, and study designs, but the overall trend was for a reduced risk for recent-onset AF with RAAS blockers, particularly in high-risk patients with left ventricular dysfunction and hypertrophy [74]. Using the General Practice Research Database (GPRD), a large validated UK primary care database, researchers compared relative risk estimates for incident AF among patients with hypertension who were receiving drugs from various antihypertensive drug classes [75]. A total of 4661 patients with AF and 18,642 matched control participants from a population of 682 993 patients treated for hypertension were identified. The results showed that at least 1 year of monotherapy with ACE inhibitors (odds ratio [OR], 0.75 [95% CI, 0.65 to 0.87]), ARBs (OR, 0.71 [CI, 0.57 to 0.89]), or beta-blockers (OR, 0.78 [CI, 0.67 to 0.92]) was associated with a lower risk for AF than monotherapy with CCBs. In addition to reducing elevated BP, the beneficial effects of RAAS blockers on reducing the risk for AF can be partly explained by prevention of atrial remodeling and reversal of LVH [17]. In combination, a beta-blocker and ACE inhibitor may therefore provide primary prevention of new-onset AF and secondary prevention of recurrent AF.

### Evidence Supporting Beta-Blockers

Approaches to control ventricular rate in AF were investigated in the AFFIRM trial, in which initial treatment included a beta-blocker, CCB, or digoxin as monotherapy or in combination with digoxin [76]. Overall rate control was achieved in 70% of patients prescribed beta-blockers as the initial therapy (with or without digoxin), 54% with CCBs (with or without digoxin), and 58% with digoxin alone.

A large Danish observational study used data from national registries to identify patients with non-valvular AF with or without concomitant heart failure [77]. Patients were stratified into beta-blocker users and non-users and according to the presence of a heart failure diagnosis and followed for up to 5 years. Beta-blocker therapy was associated with lower all-cause mortality in AF patients with and without concomitant heart failure; the respective hazard ratios (HR) were 0.75 (95% confidence interval [CI], 0.71–0.79) and 0.73 (95% CI, 0.71–0.76). These data suggest a potential benefit of beta-blocker treatment in patients with AF, regardless of concomitant disease, corroborating current guideline recommendations on beta-blocker use in patients with AF [4, 78]. Similar studies conducted in Asia have found evidence of a lower mortality with beta-blocker therapy in AF patients with concomitant heart failure [79, 80], although not always in patients without heart failure [80].

Analysis of a US National Health Insurance Service database in patients with concomitant AF and obstructive lung disease from 2002 to 2015 also revealed that rate control treatment with either cardio-selective or nonselective beta-blockers was associated with a lower risk of mortality (HR 0.84; 95% CI, 0.75–0.94; *P* = 0.002 and HR 0.85; 95% CI, 0.77–0.95; *P* = 0.003, respectively) compared with the use of CCBs [81]. Digoxin use was related with worse survival, with marginal statistical significance (HR 1.09; 95% CI, 1.00–1.18; *P* = 0.053). Prospective randomized trials are now required to confirm these findings. Current guidelines recommend that a beta-blocker or non-dihydropyridine CCB should be considered a part of the treatment of hypertension if rate control is required [4].

## Heart Failure

In the CVD continuum, heart failure is not regarded as a discrete clinical entity, but as the endpoint in the chain of CVD events. Myocardial ischemia and the resultant ventricular remodeling cause left ventricular function to deteriorate, a process that may ultimately lead to heart failure [82]. The progression from hypertension to heart failure through the CVD continuum involves several pathophysiological processes involving both the SNS and RAAS. A better understanding of these pathophysiological mechanisms has led to the use of agents that can intervene at different stages of the continuum. Treatment of heart failure with reduced left ventricular ejection fraction (HFrEF) is directed toward alleviating symptoms, hemodynamic stabilization, and addressing the underlying condition [83]. A beta-blocker is recommended in addition to an ACE inhibitor for symptomatic patients with HFrEF to reduce the risk of heart failure hospitalization and death [83]; a mineralocorticoid/aldosterone receptor antagonist may be added in those who remain symptomatic despite treatment with a beta-blocker and ACE inhibitor. Recent data also suggest that an angiotensin receptor-neprilysin inhibitor (ARNI) is beneficial for improving health status in patients with HFrEF [84]. Diuretics are effective in those with signs and symptoms of volume overload (pulmonary congestion, peripheral edema). For select patients, for instance, those with an abnormal QRS duration, device therapy such as chronic resynchronization therapy can be combined with optimal medical therapy. When added to current standard drugs in patients with HFrEF, the sodium-glucose co-transporter 2 (SGLT-2) inhibitors dapagliflozin and empagliflozin have shown clinically relevant reductions in mortality and heart failure hospitalizations, as well as improvements in quality of life both in patients with type 2 diabetes and those without [85, 86]. In a recent update from the Heart Failure Association of the ESC, dapagliflozin or empagliflozin is recommended to reduce the risk of heart failure hospitalization and cardiovascular death in HFrEF patients already receiving guideline-directed medical therapy, regardless of the presence of type 2 diabetes [87].

### Evidence Supporting ACE Inhibitors

ACE inhibitors have been shown to reduce mortality and morbidity in patients with HFrEF and are recommended, unless contraindicated or not tolerated, in all symptomatic patients [4, 5]. The CONSENSUS trial compared enalapril with placebo in addition to standard care in patients with severe HFrEF (NYHA class IV symptoms). The primary endpoints were 6-month mortality and cause of death [88]. Mortality was reduced by 40% at 6 months (*P* = 0.002) on active treatment and by 31% at 1 year (*P* = 0.001). The beneficial effect on mortality was due to a reduction in death from the progression of heart failure. The SOLVD trial recruited all patients with HFrEF, regardless of their NYHA classification, and randomized 2,569 patients to enalapril or placebo [89]. Compared with placebo, patients in the enalapril group had a significantly lower rate of mortality (35.2% vs. 39.7%; *P* < 0.0036), deaths due to progressive heart failure or arrhythmia (16.3% vs. 19.5%; *P* < 0.0045), and frequency of hospitalizations for heart failure (25.8% vs. 36.6%; *P* < 0.001). Post hoc sub-group analyses showed that enalapril was beneficial in all four NYHA classes of heart failure. ARBs have not been consistently proven to reduce mortality in patients with HFrEF, and their use should be restricted to patients intolerant of an ACE inhibitor.

### Evidence Supporting Beta-Blockers

Beta-blockers are effective in heart failure by their actions to slow heart rate and reduce myocardial contractility, and several large-scale, placebo-controlled beta-blocker trials have been conducted. CIBIS II (Cardiac Insufficiency Bisoprolol Study II) was the first to show a mortality benefit in moderate-to-severe stable HFrEF (NYHA III or IV; LVEF ≤ 35%) [90]. This was followed by the MERIT-HF (Metoprolol CR/XL Randomized Intervention Trial in Congestive Heart Failure) in mild-to-moderate, stable, HFrEF [91] and the COPERNICUS (Carvedilol Prospective Randomized Cumulative Survival) trial in severe, stable, HFrEF [92]. In all three trials, the reduction in all-cause mortality was in the region of 34–35%. The SENIORS (Study of the Effects of Nebivolol Intervention on Outcomes and Rehospitalisation in Seniors with Heart Failure) trial in elderly patients, differed from the above in that it also included patients with HFpEF as well as a primary endpoint that included hospitalization due to cardiovascular causes in addition to all-cause mortality. SENIORS showed a significant reduction of 14% in its composite primary endpoint [93].

The above trials required patients to already be on optimal medical therapy, and as this included an ACE inhibitor, these were typically initiated first. CIBIS III evaluated whether the reverse was equally effective. In this open-label, non-inferiority trial, 1010 patients aged ≥ 65 years with stable New York Heart Association class II or III heart failure and LVEF ≤ 35% received initial monotherapy with either bisoprolol or enalapril for 6 months, followed by their combination for 6 to 24 months. Both strategies showed similar efficacy for the combined primary endpoint of mortality or all-cause hospitalization. The bisoprolol-first approach was associated with a significant reduction in sudden death of 46% during the first year of treatment (*P* < 0.05) compared with ACE inhibitor first [94]. The data support selection of either agent as initial therapy for stable, mild-to-moderate chronic heart failure and suggest that early beta-blocker therapy reduces the risk of sudden death in the first year. Collectively, these trials indicate that beta-blockers and ACE inhibitors, when taken concurrently with other HFrEF medications, provide significant reductions in morbidity and mortality and are recommended for the treatment of every patient with HFrEF, unless contraindicated or not tolerated [83]. The potential benefits of beta-blockers in HFpEF continue to be explored [95].

## Synergistic Neuroendocrine Blockade

The use of a beta-blocker and ACE inhibitor provides a comprehensive neuroendocrine blockade. The beta-blocker component targets hypertension driven by the SNS, with cardiovascular beta-adrenergic blockade reducing cardiac output. The ACE inhibitor component acts on the renin-angiotensin system to induce vasodilation and reduce vascular resistance**.** Bisoprolol and perindopril are two representative first-line therapies with complementary mechanisms of action and strong outcome data in several high-risk groups. Bisoprolol is a cardioselective beta-blocker, with a 19-fold higher affinity for the beta-1 receptor than for the beta-2 receptor [96], and has a 24 h duration of action [97]. In the ACE inhibitor class, perindopril has the highest selectivity for bradykinin versus angiotensin I binding sites [98]. It has a 24 h duration of action, and trough effects are about 87–100% of peak effects [99].

**Fig. 2:**
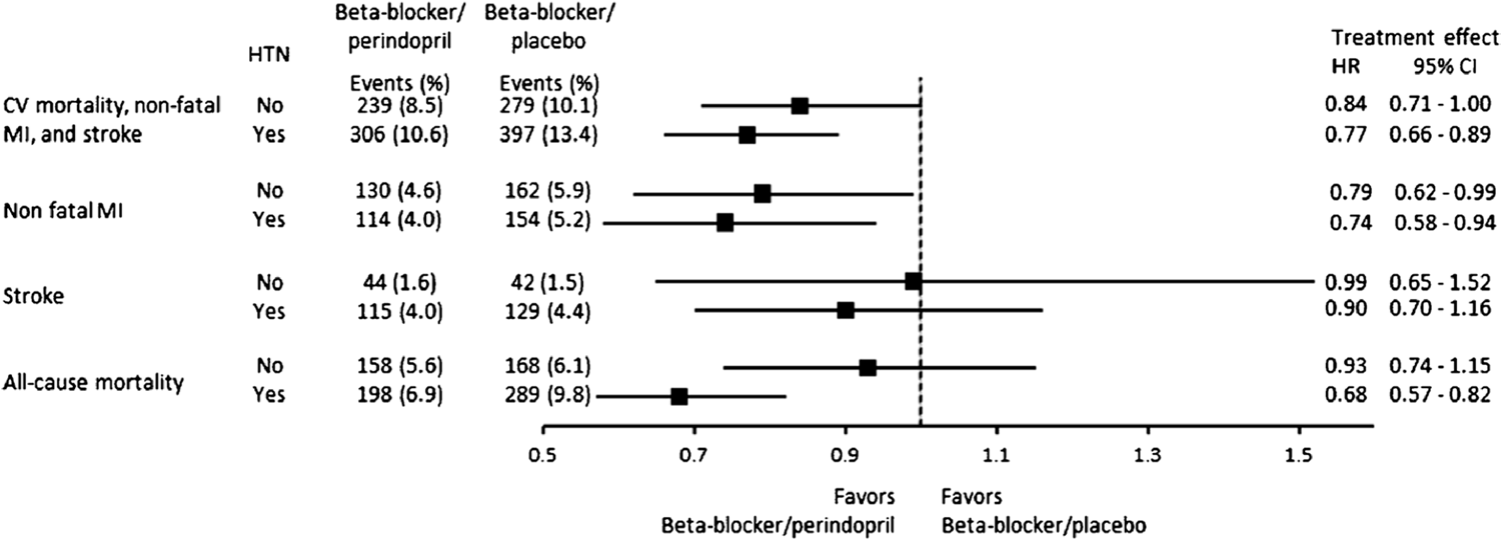
Data from a retrospective pooled analysis of patients from three large perindopril outcome trials (EUROPA, ADVANCE, and PROGRESS) who received perindopril or placebo and were already on beta-blocker therapy. Adding perindopril to a beta-blocker treatment was associated with a decreased risk of the primary composite endpoint of cardiovascular mortality, non-fatal myocardial infarction, and stroke, as well as the secondary endpoints of non-fatal myocardial infarction and all-cause mortality ( reproduced from Brugts et al., 2017 [101] (published under CC-BY license)

Evidence for the benefits of perindopril in combination with a beta-blocker come from the EUROPA trial, which randomized over 12,000 patients with stable CAD to perindopril or placebo and which included patients with angina or a previous myocardial infarction [48]. At study entry, 62% of the randomized patients were on beta-blockers. In these patients, the addition of perindopril to the beta-blocker produced a 24% reduction in relative risk of the combined primary endpoint (cardiovascular death, non-fatal myocardial infarction, and resuscitated cardiac arrest) compared with the beta-blocker/placebo group [100]. These data have been extended by a retrospective pooled analysis of patients with vascular disease from three large perindopril outcome trials (EUROPA, ADVANCE, and PROGRESS) who received perindopril or placebo and were already on beta-blocker therapy. Among the 11,418 patients taking a beta-blocker, 5700 were randomized to a perindopril-based regimen and 5718 to placebo [101]. Adding perindopril to a beta-blocker treatment was associated with a decreased risk of the primary composite endpoint of cardiovascular mortality, non-fatal myocardial infarction, and stroke, as well as the secondary endpoints of non-fatal myocardial infarction and all-cause mortality (Fig. 2).

## Single-Pill Delivery of Bisoprolol and Perindopril

The first single-pill combination of a beta-blocker and ACE inhibitor became available in 2016 in the form of bisoprolol/perindopril (Cosyrel®) and is indicated in arterial hypertension, stable CAD, and/or heart failure.

Data supporting the benefits of bisoprolol/perindopril administered as a single-pill combination come from a study completed for 2394 patients, which examined whether heart rate and BP were improved when patients with CAD and hypertension who had previously received bisoprolol were prescribed the fixed-dose combination [102]. After 4 weeks of treatment, 84.9% of patients achieved a heart rate ≤ 70 bpm and 86.9% a BP ≤ 140/90 mmHg. The combination also decreased the frequency of angina attacks and improved treatment adherence.

The addition of the same single-pill combination to the treatment regimen of patients after revascularization for an acute coronary syndrome (a very high-risk population) also led to achievement of target BP and heart rate levels within 1 month of starting therapy and stabilization of these hemodynamic values and clinical symptoms by 3 months [103]. As a result, patients could be included in cardiac rehabilitation programs.

## Summary

There is robust evidence on the benefits of beta-blocker and ACE inhibitor use in patients with hypertension and elevated heart rate, CAD, AF, and heart failure. In combination, these two classes provide a comprehensive neuroendocrine blockade targeting both the heart, where beta blockade reduces cardiac output, and the vessels, where ACE inhibition induces vasodilation among other actions. The benefits of a bisoprolol/perindopril combination are supported by a large evidence base of use confirming their well-established, long-term efficacy and tolerability. Each component has a long elimination half-life providing 24-h efficacy with once-daily administration. The available data suggest that combining these agents into a single pill would provide a valuable treatment option for a number of patient profiles allowing them to more rapidly achieve target heart rate and blood pressure levels at the same time as reducing cardiac events. Such an approach is widely supported by clinical trials and associated clinical guidelines and represents a positive step forward in patient care.

## Data Availability

All data and materials submitted with the paper.
